# Supplementary material for: Enhanced piezoelectricity from highly polarizable oriented amorphous fractions in biaxially oriented poly(vinylidene fluoride) with pure β crystals
Source: Nat Commun. 2021 Jan 29;12:675. doi: 10.1038/s41467-020-20662-7 (PMC7846586; doi:10.1038/s41467-020-20662-7)
Supplement: Supplementary file 1 — Supplementary Information [file 41467_2020_20662_MOESM1_ESM.pdf]

Supplementary Information

Enhanced piezoelectricity from highly polarizable oriented amorphous fractions in biaxially oriented poly(vinylidene fluoride) with pure β crystals

Yanfei Huang[1,#], Guanchun Rui[2,#], Qiong Li[2], Elshad Allahyarov[3,4,5], Ruipeng Li[6], Masafumi Fukuto[6], Gan-Ji Zhong[7], Jia-Zhuang Xu[7], Zhong-Ming Li[7], Philip L. Taylor[3,*], Lei Zhu[2,*]

[1] *College of Materials Science and Engineering, Shenzhen Key Laboratory of Polymer Science and Technology, Shenzhen University, Shenzhen 518055, P. R. China*
[2] *Department of Macromolecular Science and Engineering, Case Western Reserve University, Cleveland, Ohio 44106-7202, United States*
[3] *Department of Physics, Case Western Reserve University, Cleveland, Ohio 44106, United States*
[4] *Theoretische Chemie, Universität Duisburg-Essen, D-45141 Essen, Germany*
[5] *Theoretical Department, Joint Institute for High Temperatures, Russian Academy of Sciences, 13/19 Izhorskaya street, Moscow 125412, Russia*
[6] *National Synchrotron Light Source II, Brookhaven National Laboratory, Upton, New York 11973, United States*
[7] *College of Polymer Science and Engineering, State Key Laboratory of Polymer Materials Engineering, Sichuan University, Chengdu 610065, Sichuan, P. R. China*

# These authors contributed equally to this work: Yanfei Huang, Guanchun Rui

* Corresponding authors. Emails: lxz121@case.edu (L. Zhu) and plt@case.edu (P.L. Taylor)

Table of Content

Note 1. Additional 2D WAXD patterns for the fresh and poled BOPVDF films
Note 2. Subtraction of AC electronic conduction from D-E loops for the poled BOPVDF
Note 3. Subtraction of the deformational polarization
Note 4. Frequency-scan BDS results for poled BOPVDF
Note 5. Determination of permanent remanent polarization P_{r0} by subtracting the "relaxed polarization" (P_r^u)
Note 6. Setup of the piezoelectric measurements
Note 7. Direct piezoelectric measurements of d_{33}, d_{31}, and d_{32} for the fresh BOPVDF
Note 8. Direct piezoelectric charge measurements for the poled BOPVDF
Note 9. Determination of the accuracy of the direct piezoelectric measurement
Note 10. Estimation of individual layer thicknesses from SAXS study
Note 11. Molecular dynamics (MD) simulation
References

Supplementary Note 1. Additional 2D WAXD patterns for the fresh and poled BOPVDF films

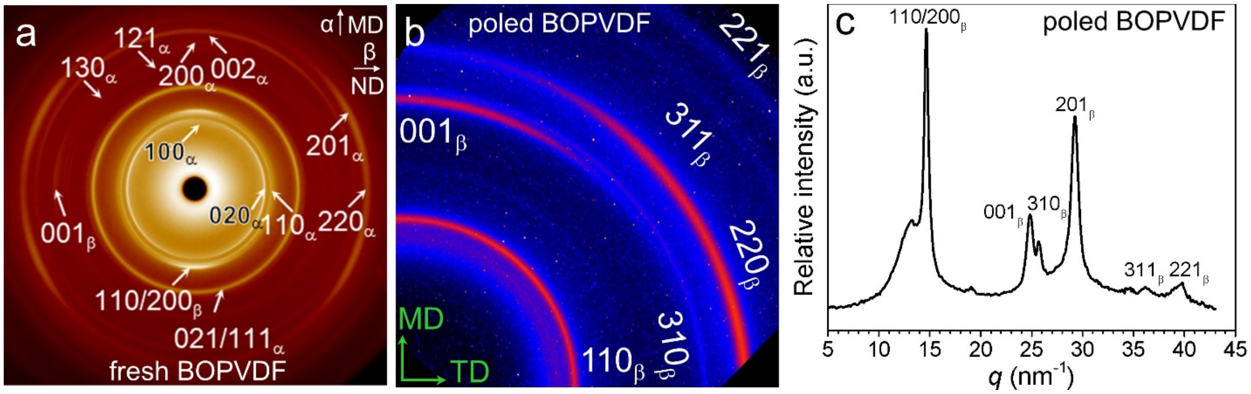

Supplementary Fig. 1. (a) 2D edge-on WAXD pattern for the fresh BOPVDF at room temperature. The X-ray beam is along the transverse direction (TD) with machine direction (MD) vertical and film normal direction (ND) horizontal. Major reflections for both α and β phases are shown. Based on the crystal orientation analysis, the *c*-axis of the major α crystals is oriented primarily in the MD and the *c*-axis of the minor β crystals is orientated in the ND. (b) 2D flat-on WAXD pattern for the poled BOPVDF at room temperature. The X-ray beam is along the ND with MD vertical and TD horizontal. Major reflections for the neat β phase are shown, from which the *c*-axis of the β crystals is oriented primarily along the MD. (c) Integrated 1D WAXD profile for the 2D pattern in (b). Major reflections are labelled.

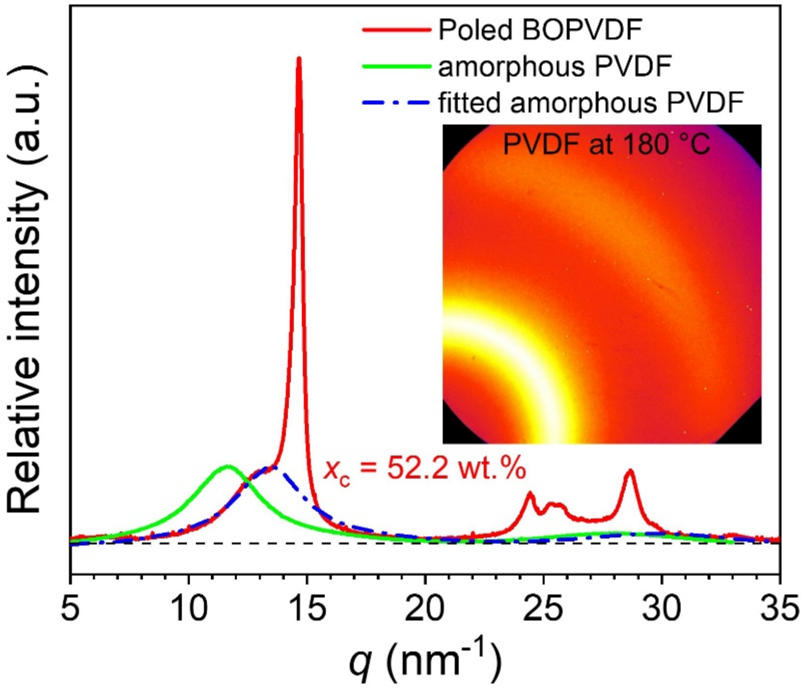

Supplementary Fig. 2. Estimation of the crystallinity from the 1D WAXD profile of the poled BOPVDF film with a pure β phase. The green curve shows the 1D WAXD profile obtained from the inset 2D WAXD pattern for PVDF at 180 °C. The blue (dash-dot) curve shows the fitted amorphous halo for the amorphous phase in the poled BOPVDF film. The crystallinity is estimated to be ~0.52.

Because the heat of fusion for 100% extended-chain β crystals is not available, we cannot use DSC to determine its crystallinity. Instead, 1D WAXD was used to determine the crystallinity, as shown in Supplementary Fig. 2. After fitting the amorphous halo (by shifting the amorphous PVDF WAXD profile at 180 °C), the crystallinity was estimated to be 0.52. This value is similar to that (0.54) reported for the fresh BOPVDF before.[1]

Meanwhile, it is interesting to see that the amorphous halo of the molten PVDF at 180 °C showed an average interchain spacing of 0.537 nm. For the poled BOPVDF at room temperature, the fitted amorphous halo had an average interchain spacing of 0.467 nm. This difference could not be accounted for by thermal expansion alone. Instead, as discussed in the main text, BOPVDF contained a significant amount of oriented amorphous fraction (OAF, $f_{OAF} > 0.25$), which grew out from the crystal basal planes. As a result, the average distance between chains in OAF must be smaller than those in the truly isotropic amorphous fraction (IAF).

Supplementary Note 2. Subtraction of AC electronic conduction from D-E loops for the poled BOPVDF

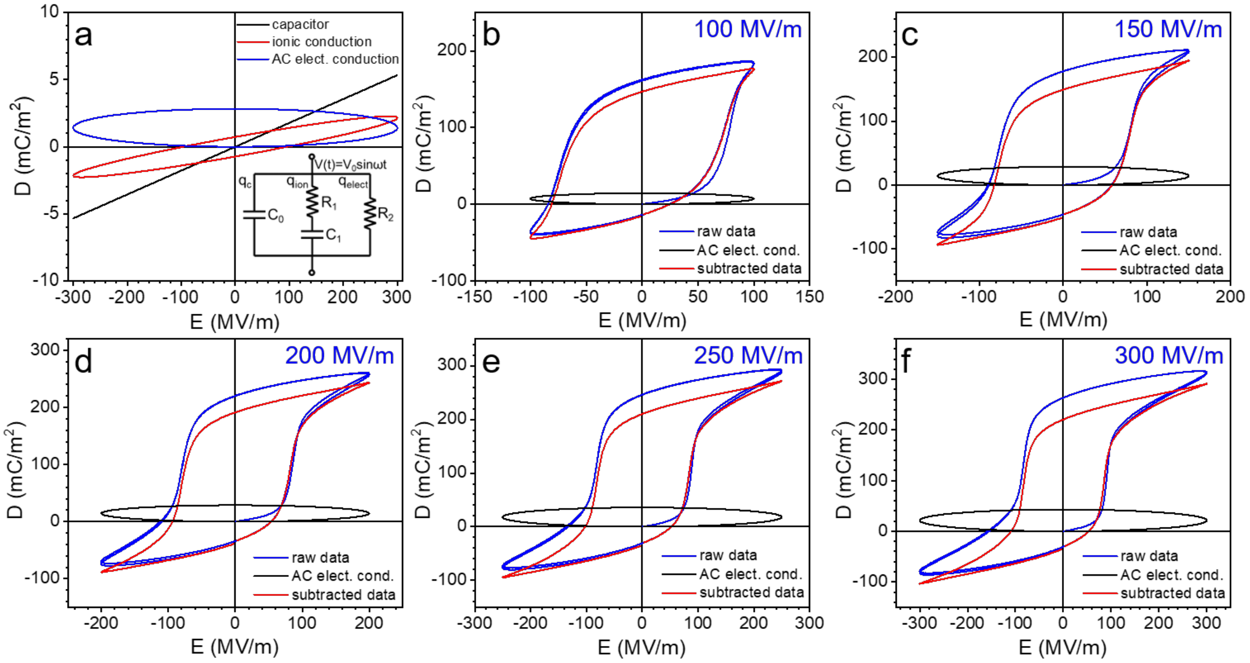

Supplementary Fig. 3. (a) A real polymer film capacitor can be simulated by an equivalent circuit (inset) with parallel capacitor (C_0), series R_1 and C_1 for ionic conduction, and resister (R_2) for AC electronic conduction. Their D-E loops are calculated from the charges in each branch under a sinusoidal driving voltage, $V(t) = V_0 \sin\omega t$, as reported before.[3] (b-f) show raw data, fitted AC electronic conduction, and subtracted data for bipolar D-E loops for the highly poled BOPVDF film at room temperature.

For the highly poled BOPVDF, AC electronic conduction became more significant when compared to the fresh BOPVDF. We speculate that injected real charges (electrons and/or holes) under high poling fields might be the reason for increased AC electronic conduction for the poled BOPVDF film. Note that these injected charges do not need to penetrate through the entire thickness of the film, like the leakage current. Instead, charge-injection and recombination at the sample-electrode interfaces could cause the electronic conduction loss, as reported before.[2]

Therefore, we call it AC electronic conduction. It is necessary to subtract AC electronic conduction from the bipolar D-E loops using the method introduced in our previous publications.[3]

An equivalent RC circuit (see the inset of Supplementary Fig. 3a) consists of i) pure capacitor C_0, ii) lossy capacitor with R_1 and C_1 in series, and iii) AC conduction loss R_2. The lossy capacitor can represent the impurity ion conduction in PVDF. A sinusoidal AC external voltage with the amplitude of V_0 is applied to the circuit, $V(t) = V_0\sin(\omega t)$, where ω is the angular frequency ($\omega = 2\pi f$ with f being the frequency). The Q_0, Q_1, and Q_2 are the charges passing through three shunt circuits at a certain time t. The equations for calculating these three charges are:

$$Q_0 = C_0 V_0 \sin(\omega t)$$ (Supplementary Eqn. 1)

$$Q_1 = \frac{V_0 C_1}{1+(C_1 R_1 \omega)^2}\sin(\omega t) - \frac{\omega R_1 V_0 C_1^2}{1+(C_1 R_1 \omega)^2}\cos(\omega t)$$ (Supplementary Eqn. 2)

$$Q_2 = \frac{V_0}{\omega R_2}[1 - \cos(\omega t)]$$ (Supplementary Eqn. 3)

The electric displacement can be defined as $D = Q/S$ (S is the surface area of the electrode) and the applied electric field $E = V/d = V_0 \sin(\omega t)/d$ with d being the thickness of the film between two electrodes. The D and E values for the dielectric capacitor, lossy capacitor, and AC electronic conduction contributions could be calculated based on the above equations. The D-E loops from individual contributions are shown in Supplementary Fig. 3a with the following parameters: $f = 10$ Hz, $d = 20$ μm, $S = 0.045$ cm^2, $V_0 = 6000$ V, $C_0 = 4$ pF, $C_1 = 1.8$ pF, $R_1 = 3$ GΩ, and $R_2 = 15$ GΩ. These parameters are chosen to clearly represent separate contributions from pure capacitor, ionic conduction, and AC electronic conduction (Supplementary Fig. 3a). After subtraction of the AC electronic conduction, all ferroelectric D-E loops are centered in Fig. 2 of the main text.

Supplementary Note 3. Subtraction of linear deformational polarization

As reported in our previous work,[4] the deformational polarization (D_{def}) cannot be directly measured for the neat BOPVDF film because of ferroelectric switching above the E_c at 70 MV/m. For polycarbonate (PC)/PVDF multilayer films, ferroelectric switching in PVDF can be effectively prohibited. Therefore, we are able to extract the linear deformational D-E loops for neat PVDF from the D-E loops of the PC/PVDF multilayer films. In this study, we used a PC/PVDF 50/50 (vol./vol.) bilayer film for the extraction. The theoretical derivation is described below.

For a bilayer polymer film with equal layer thickness, the nominal electric field for layer 1 (E_1) and layer 2 (E_2) obeys the following relationship:

$$E_2 = \frac{2\varepsilon_{r1} E_0}{\varepsilon_{r2} + \varepsilon_{r1}}$$ (Supplementary Eqn. 4)

where E_0 is the applied electric field on the bilayer film, and ε_{r1} and ε_{r2} are the relative permittivities of PC (2.9) and PVDF, respectively. The detailed deduction of Supplementary Eqn. 4 is shown in our previous report.[4] Since PC is a linear dielectric polymer, its dielectric constant does not change with electric field and frequency at room temperature. However, PVDF is a nonlinear dielectric (i.e., ferroelectric) polymer, its ε_{r2} may vary with electric field and frequency, even at room temperature. As reported in our previous work,[4] the ε_{r2} for PVDF can be obtained from the experimentally measured electric displacement (D) of the bilayer PC/PVDF film:

$$\varepsilon_{r2} = \frac{\varepsilon_{r1} D}{2\varepsilon_0 \varepsilon_{r1} - D}$$ (Supplementary Eqn. 5)

From Eqns. S4 and S5, we obtain:

$$E_2 = 2E_0 - \frac{D}{\varepsilon_{r1}\varepsilon_0} \qquad \text{(Supplementary Eqn. 6)}$$

By plotting D versus E_2, we can extract the deformational D-E loops for neat PVDF. Results are shown in Fig. 2b.

Supplementary Note 4. Frequency-scan BDS results for poled BOPVDF

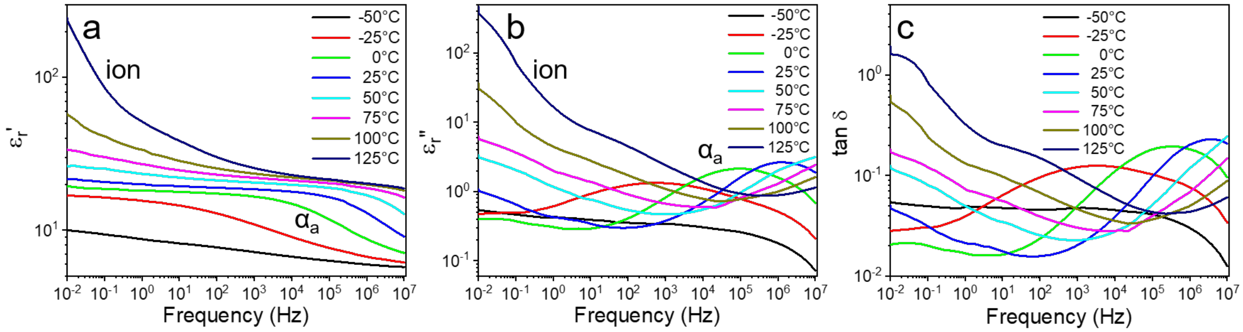

Supplementary Fig. 4. Frequency-scan BDS results of (a) ε_r', (b) ε_r'', and (c) tanδ for the highly poled BOPVDF film at different temperatures, ranging from -50 to 125 °C. Note that the glass transition temperature (T_g) is -39 °C.

The high dissipation factor for the poled BOPVDF is attributed to the dielectric nonlinearity caused by the high P_{r0},[4,5] and cannot be minimized without decreasing P_{r0} and the piezoelectric property. However, this low-field dissipation will not significantly increase the hysteresis loop loss for piezoelectricity, because the D-E loop is rather linear and narrow when the poling electric field is below 50 MV/m (see Fig. 2d in the main text).

Supplementary Note 5. Determination of permanent remanent polarization P_{r0} by subtracting the "relaxed polarization" (P_r^u)

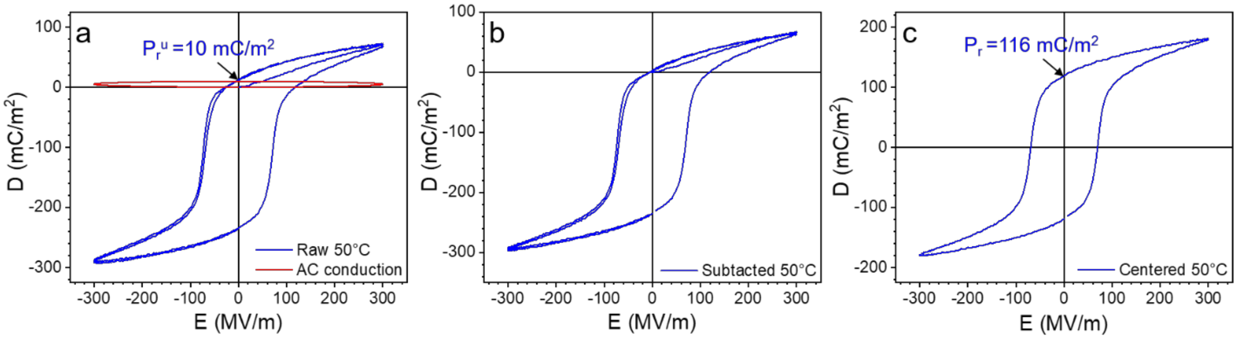

Supplementary Fig. 5. (a) Raw first two bipolar D-E loops for the freshly poled BOPVDF at 50 °C. The poling frequency was 10 Hz with a sinusoidal waveform. Meanwhile, the horizontal AC electronic conduction loop is shown. (b) After subtracting the AC electronic conduction loop, neat ferroelectric switching loops are obtained. From the centered second D-E loop in (c), the instantaneous remanent polarization (P_r) is obtained.

To determine the permanent remanent polarization (P_{r0}), the freshly poled BOPVDF film was thermally annealed at an elevated temperature in a heated silicone oil bath for 30 min. While keeping the same polarity, the first two continuous bipolar D-E loops at 300 MV/m and 10 Hz were recorded at that temperature. An example at 50 °C is shown in Supplementary Fig. 5a. From the first half unipolar loop, the remanent (or relaxed) polarization (P_r^u) was determined to be 10 mC/m^2. For the second loop, the AC electronic conduction was determined as the red horizontal loop. By subtracting the AC electronic conduction from the raw D-E loops, the neat ferroelectric loops were obtained, as shown in Supplementary Fig. 5b. The second bipolar loop was centered in Supplementary Fig. 5c, from which the instantaneous remanent polarization (P_r) was determined to be 116 mC/m^2. The P_{r0} can be determined as: $P_r - P_r^u = 106$ mC/m^2. The same procedure was repeated for other temperatures up to 125 °C. This approach is modified from the positive-up and negative-down (PUND) method reported in the literature.[6]

Supplementary Note 6. Setup of the piezoelectric measurements

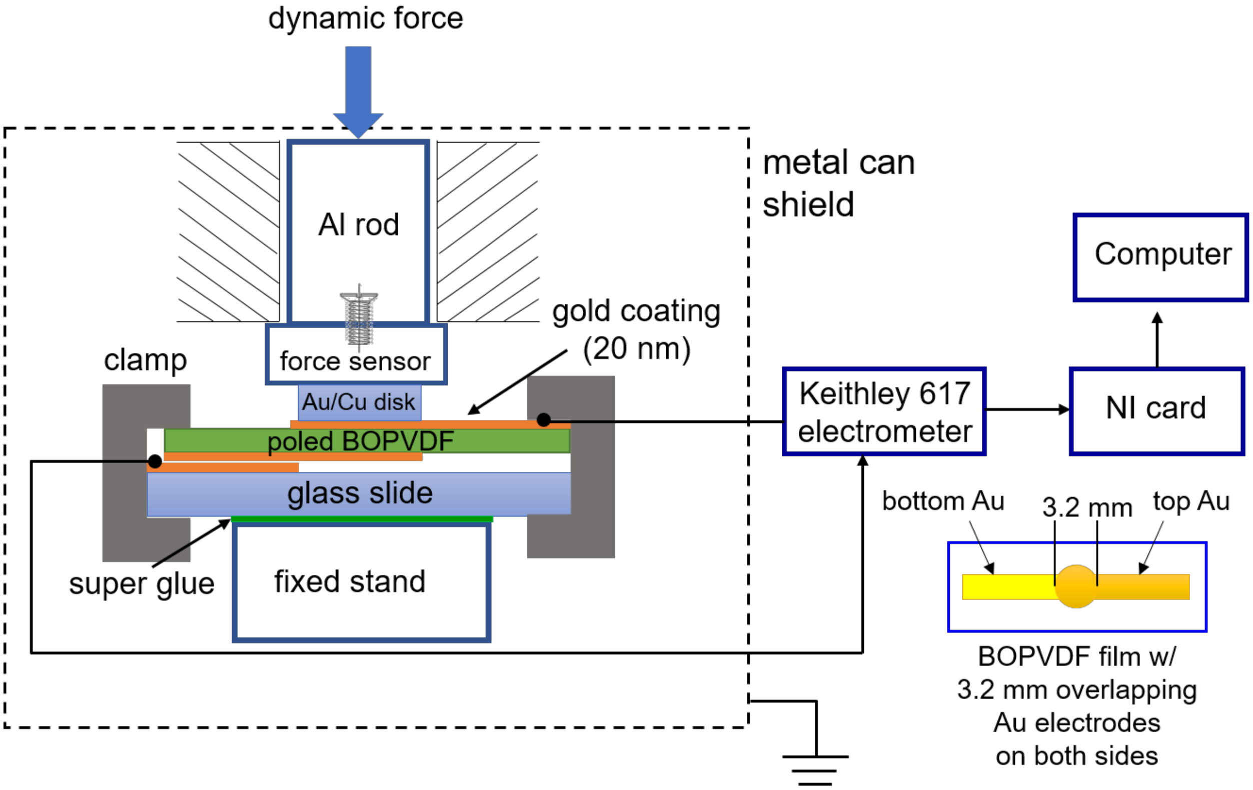

Supplementary Fig. 6. Scheme of the out-of-plane piezoelectric coefficient, d_{33}, measurement setup. The sample geometry with gold-coatings on both sides is also shown.

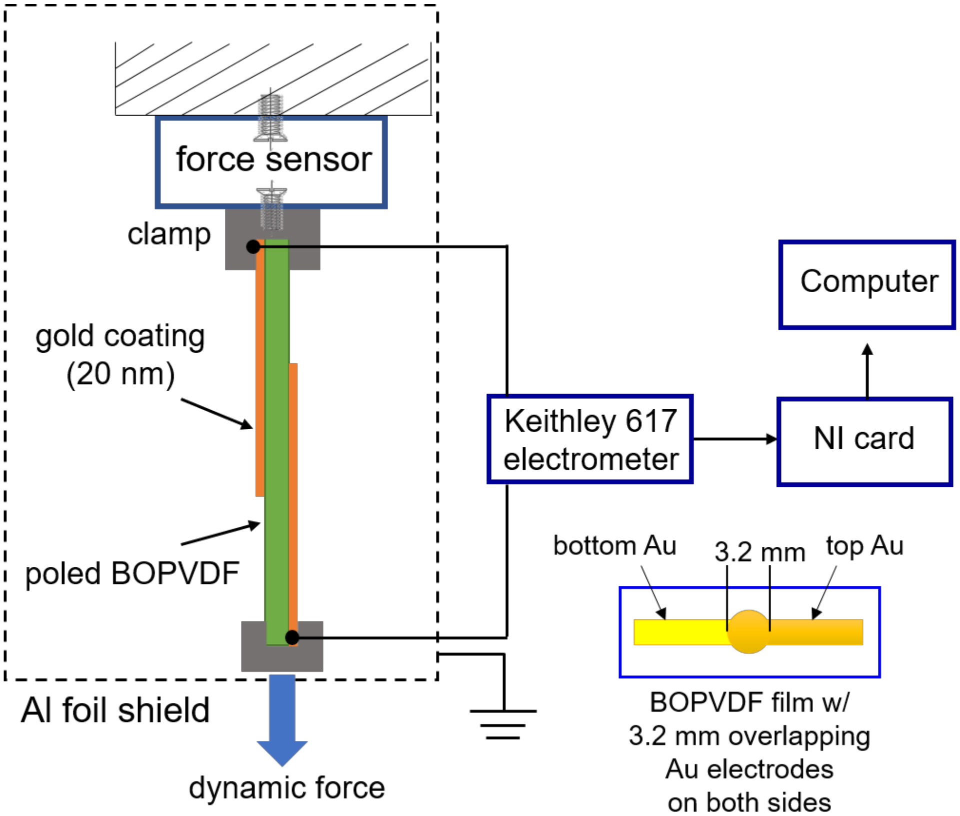

Supplementary Fig. 7. Scheme of the in-plane piezoelectric coefficient, d_{31} and d_{32}, measurement setup. The sample geometry with gold-coatings on both sides is also shown.

The fresh BOPVDF films with the same sample geometry as the poled BOPVDF films were prepared as the control. Their d_{33}, d_{31}, and d_{32} were also measured in the same way as reported in Methods in the main text. Since the fresh BOPVDF with randomly oriented dipoles/domains should not show any piezoelectricity, if any charges had been generated from the direct piezoelectric tests, they could only be induced by triboelectricity. From our control experiments, no charges were generated during the direct piezoelectric tests (see results later); therefore, we conclude that triboelectricity was successfully avoided during our direct piezoelectric tests.

To ensure the accuracy of our direct piezoelectric measurements, a commercial piezoelectric ceramic, lead zirconate titanate (PZT, Navy VI, APC International, Ltd., USA), was tested using the home-made setup. The PZT disk had a diameter of 10 mm, thickness of 1 mm, and reported d_{33} of 630 pC/N. The measurement set-up is the same as Supplementary Fig. 6, except that the 3.2 mm Au-coated Cu disk was replaced by a 10 mm Au-coated Cu disk to assure a uniform applied force. The average d_{33} was determined to be 640±10 pC/N.

Supplementary Note 7. Direct piezoelectric measurements of d_{33}, d_{31}, and d_{32} for the fresh BOPVDF

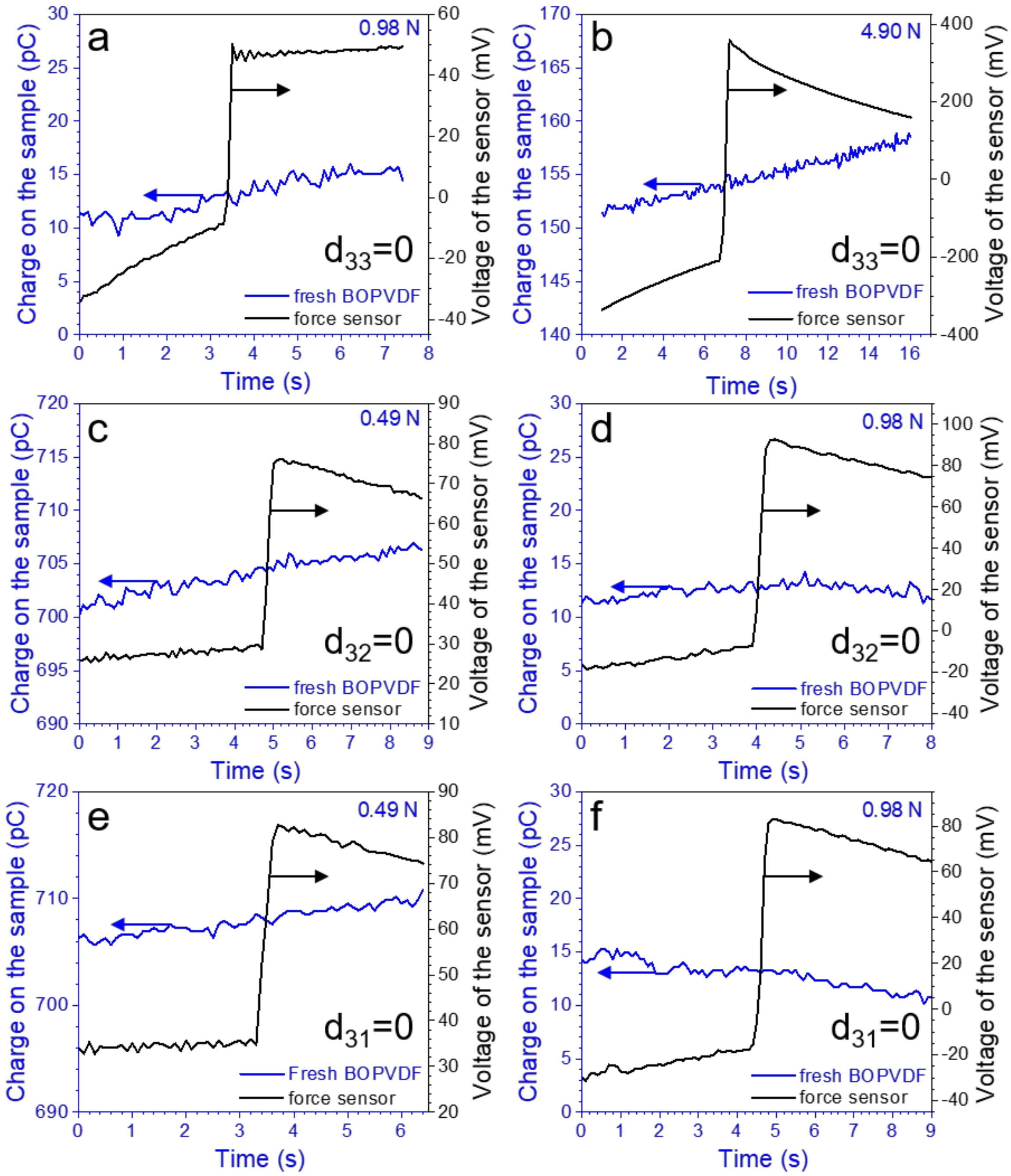

Supplementary Fig. 8. Direct piezoelectric measurements for the unpoled, fresh BOPVDF film: d_{33} under (a) 0.98 N and (b) 4.90 N; d_{32} under (c) 0.49 N and (d) 0.98 N; d_{31} under (e) 0.49 N and (f) 0.98 N. Because the permanent remanent polarization (P_{r0}) is zero, all piezoelectric coefficients are zero. Note that the charge readings are the raw experimental data. This also proves that no triboelectricity is present for our d_{33} measurement setup in Supplementary Fig. 6.

Supplementary Note 8. Direct piezoelectric charge measurements for the poled BOPVDF

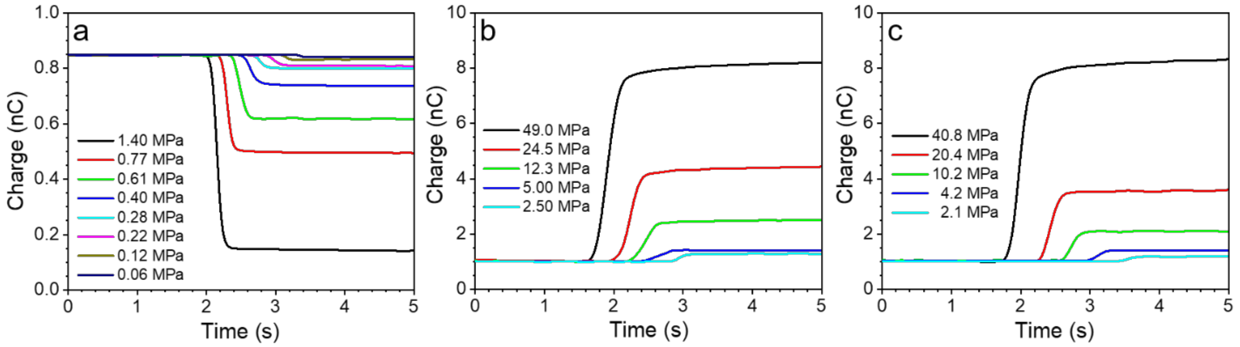

Supplementary Fig. 9. Direct piezoelectric charge measurements of (a) d_{33}, (b) d_{32}, and (c) d_{31} for the poled BOPVDF. A Keithley 617 electrometer is used to record the generated charges. Note that the dynamic stresses in the d_{33} measurement were smaller than those in the d_{32} and d_{31} measurements. Therefore, the charge generated in the d_{33} measurement was smaller.

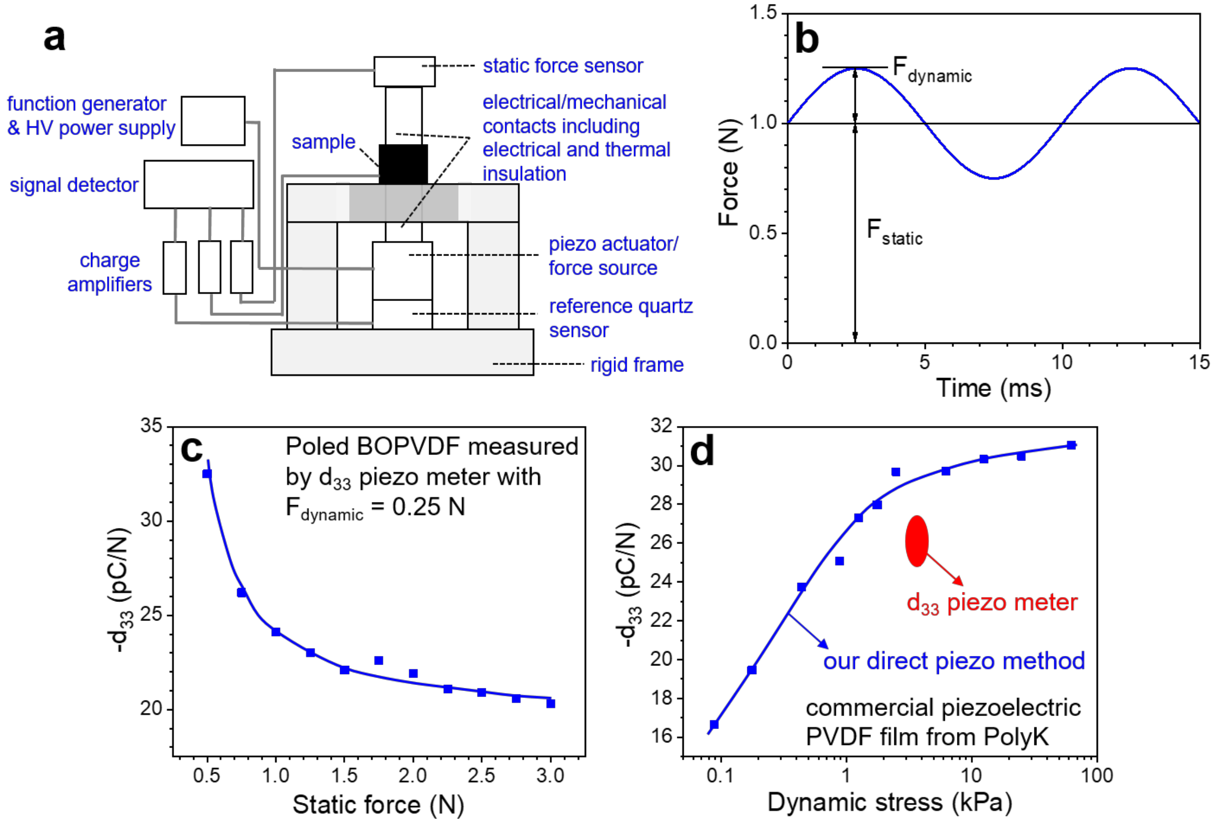

Supplementary Fig. 10. (a) Schematic of the d_{33} piezo meter using the quasi-static Berlincourt method. (b) The static (F_{static}) and dynamic force ($F_{dynamic}$) profiles applied to the sample. Usually, F_{static} should be slightly larger than $F_{dynamic}$ to avoid negative total force. The typical frequency is 100 Hz during measurement. (c) d_{33} of the poled BOPVDF as a function of F_{static} with a fixed $F_{dynamic}$ (0.25 N), measured by our d_{33} piezo meter. (d) d_{33} of the commercial piezoelectric PVDF film (120 μm, PolyK) as a function of $F_{dynamic}$, measured by our direct piezoelectric method. The d_{33} value measured by the d_{33} piezo meter (the red ellipsoid) is shown for comparison. The difference between two methods is around 10%.

Supplementary Note 9. Determination of the accuracy of the direct piezoelectric measurement

To determine the accuracy of our measurements, a commercial d_{33} meter (PKD3-2000, PolyK Technologies, State College, PA, USA) was employed to compare the results. Supplementary Fig. 10a shows the working mechanism of the Berlincourt method. At the very bottom of the piezo meter, there is a reference quartz sensor, on top of which we have a piezo actuator to generate the dynamic force [$F_{dynamic}(t)$ with a sinusoidal waveform]. For most commercial d_{33} piezo meters, the amplitude of $F_{dynamic}(t)$ ($F_{dynamic}$) is fixed, and it is 0.25 N for our piezo meter (Supplementary Fig. 10b). This piezo actuator is driven by an HV power supply with a frequency generator at 100 Hz. On the top of the piezo actuator, the sample is sandwiched by two metal electrodes. On the very top, we have a static force (F_{static}) sensor, and the static force is applied by tightening the top screw, and is used to make sure the sample does not rattle. For our d_{33} piezo meter, the F_{static} can be adjusted from 0 to ~ 6.0 N. Since the $F_{dynamic}$ is 0.25 N, the minimum F_{static} had better to be greater than 0.25 N to avoid a negative total force. Otherwise, unreliable results are obtained at low F_{static}.

The reference quartz sensor is used to determine the $F_{dynamic}$. The amplitude of charge on the sample (Q_{sample}) is measured by the signal detector (e.g., oscilloscope). Then, the piezoelectric coefficient, d_{33}, can be directly calculated using the piezoelectricity definition:

$$P_{sample} = Q_{sample}/A = d_{33}T_3 = d_{33}(F_{dynamic}/A) \qquad \text{(Supplementary Eqn. 7)}$$

where P_{sample} is the polarization of the sample, T_3 is the dynamic stress, and A is the area of the electrode/sample contact. Note, that the F_{static} does not appear in Supplementary Eqn. 7; however, it will affect the measured d_{33} results, as we will discuss later. Since the area A is cancelled on both sides, the d_{33} can be simply calculated using the following equation:

$$d_{33} = Q_{sample}/F_{dynamic} \qquad \text{(Supplementary Eqn. 8)}$$

Because A does not appear in Supplementary Eqn. 8, we do not need to measure the electrode/sample contact area to obtain the T_3. This is why we do not require an accurate probe geometry for the piezo meter, as long as the top and bottom contact areas of the sample are the same. Also, we do not need to coat both sides of the sample with metal electrodes (if we coat the sample with metal electrodes, it is still fine). This is the advantage of this quasi-static Berlincourt method, and it is really simple to use (i.e., without coating metal electrodes).

Although the F_{static} does not appear in Supplementary Eqn. 8, it can still affect the measured d_{33} results. Since the total polarization [which is caused by the total stress, i.e., (F_{static}+$F_{dynamic}$)/A] for the piezoelectric effect is fixed, the higher the applied F_{static}, the lower Q_{sample} and thus the lower d_{33} will be obtained. This is exactly seen for our poled BOPVDF film in Supplementary Fig. 8c. This F_{static} effect is true not only for piezoelectric polymers, but also for soft piezoelectric ceramics such as PC 5H (PZT).[7]

However, the $F_{dynamic}$ effect is the opposite. Namely, when the F_{static} is kept reasonably low, the larger the $F_{dynamic}$ the higher the measured d_{33}, because the higher $F_{dynamic}$ will induce more polarization and thus a larger piezoelectric effect. This is exactly seen for our experimental results in Fig. 3 in the main text. Again, this is true not only for piezoelectric polymers, but also for piezoelectric ceramics such as PZT.[8,9]

Unfortunately, most commercial d_{33} piezo meters cannot vary $F_{dynamic}$. Therefore, we cannot verify the $F_{dynamic}$ effect on d_{33} in Fig. 3a using the commercial d_{33} piezo meter. It is worth stating that in our experiment, even though the force was generated by lifting different weights from the sample, the force change is quite fast, and it is actually a dynamic force, not a static one.

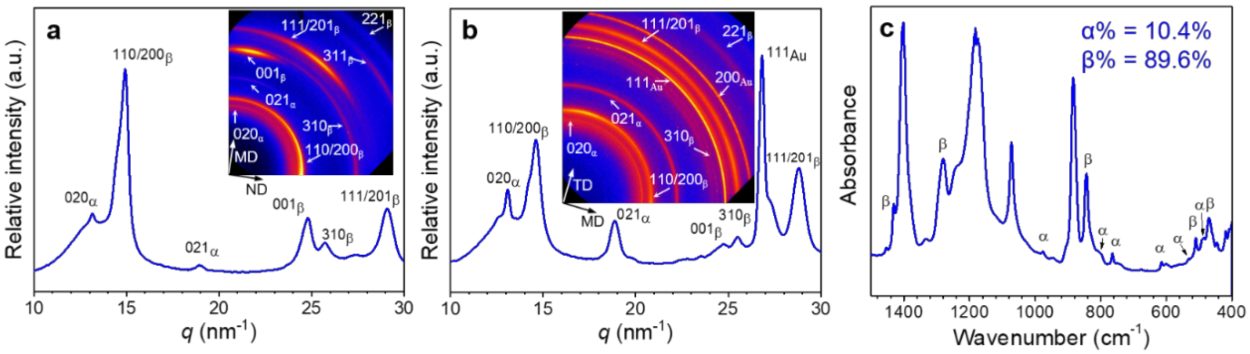

Supplementary Fig. 11. Room temperature 1D WAXD profiles for a less polarized BOPVDF film (unipolar poling at 500 MV/m for 100 times), when the X-ray is directed along (a) the TD and (b) the ND, respectively. The insets show the corresponding 2D WAXD patterns (in a logarithmic scale). The $(111)_{Au}$ reflection comes from the residual Au electrodes. (c) Transmission FTIR spectrum for the less polarized BOPVDF film at room temperature. The absorption bands for α and β crystals are labeled.

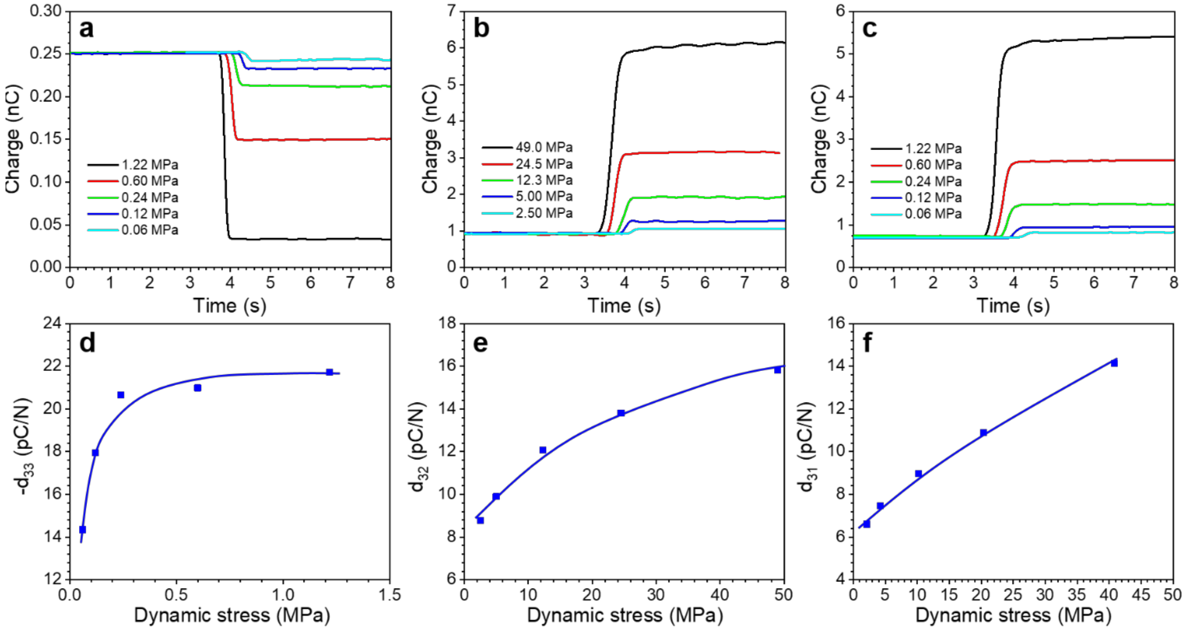

Supplementary Fig. 12. Direct piezoelectric charge measurements of (a) d_{33}, (b) d_{32}, and (c) d_{31} for the less polarized BOPVDF film (500 MV/m for 100 times). Using Eqns. 4 and 7 in Methods, direct piezoelectric coefficients are determined: (d) d_{33}, (e) d_{32}, and (f) d_{31} as a function of the dynamic stress.

Since the commercial d_{33} piezo meter cannot verify the $F_{dynamic}$-dependent d_{33}, the d_{33} of a less polarized BOPVDF (unipolar poling at 500 MV/m for 100 times) as a function of dynamic stress was determined by our direct piezoelectric charge method. Using a relative low electric field, mixed α and β phases were obtained (Supplementary Fig. 11). Using FTIR analysis (Supplementary Fig. 11c), the α and β contents are 10.4% and 89.6%, respectively. The piezoelectric properties are shown in Supplementary Fig. 12, and a lower d_{33} is obtained (Supplementary Fig. 12a,d). For d_{33}, an abrupt increase is observed below 0.3 MPa, which is somewhat consistent with the results for the highly poled BOPVDF in Fig. 3a in the main text. In

addition to d_{33}, we also measured d_{32} and d_{31}, as shown in Supplementary Fig. 12b,e and 10c,f. As in the results reported in our manuscript, d_{32} and d_{31} did not show any abrupt change as a function of dynamic stress.

Finally, we tested a commercial piezoelectric PVDF film provided by PolyK, using our direct piezoelectric method. The film was uniaxially stretched followed by corona poling to achieve a macroscopic dipole moment. The film thickness was 120 μm. As shown in Supplementary Fig. 10d, the d_{33} is also dependent upon $F_{dynamic}$; however, the d_{33} levels off around 31 pC/N. The d_{33} measured by the piezo meter is between 25 and 28 pC/N, depending on different static pre-load forces. Since $F_{dynamic}$ = 0.25 N and the electrode area A = 67.9 mm^2, the dynamic stress T_3 is estimated to be ~3.7 kPa. Comparison of the d_{33} values obtained from these two methods shows the difference to be about 10%. Therefore, we consider our measurement should be reliable.

Supplementary Note 10. Estimation of individual layer thicknesses from SAXS study

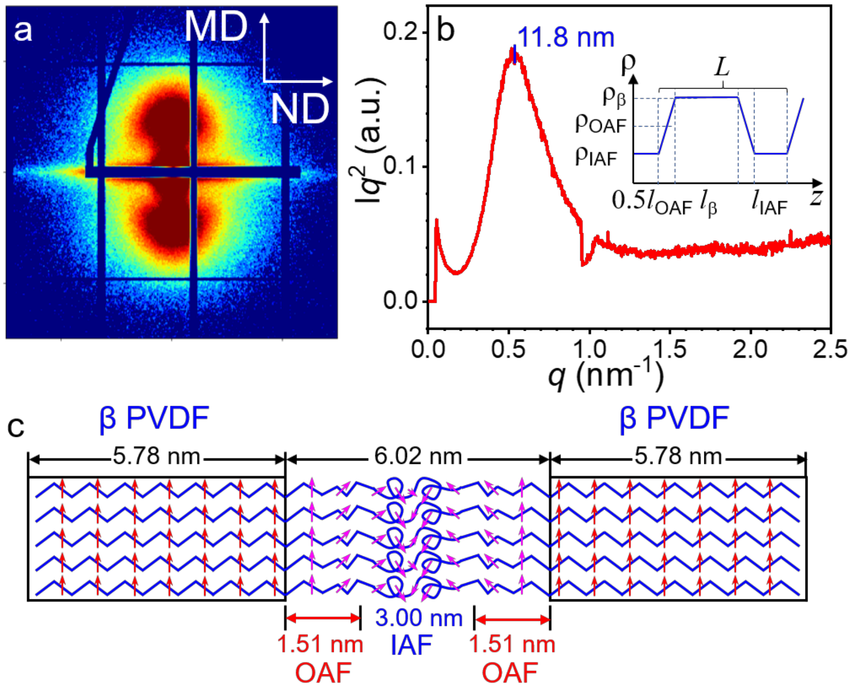

Supplementary Fig. 13. (a) 2D edge-on SAXS pattern for the poled BOPVDF. The X-ray beam is along the TD with the MD upward. (b) Lorentz-correct plot of Iq^2 versus q (I is intensity). (c) Proposed schematic representation of the three-phase model with the individual layer thicknesses calculated.

Supplementary Fig. 13a shows the 2D edge-on SAXS pattern for the poled BOPVDF. The X-ray beam was along the TD with MD in the vertical direction. From the integration, 1D SAXS profile was obtained. The Lorentz-correct plot of Iq^2 versus q (I is intensity) is shown in Supplementary Fig. 11b, where the 1D curve is integrated along the MD of the 2D SAXS pattern. From the peak value, the lamellar spacing L = 11.8 nm. The density profile of the lamellar structure is shown in the inset of Supplementary Fig. 13b. The density of the amorphous phase is ρ_{IAF} = 1.68 g/cm^3 and the density of the β crystal is ρ_β = 1.972 g/cm^3, as we reported before.[3] Then, the average density of the OAF can be calculated as $0.5(\rho_{IAF} + \rho_\beta)$ = 1.826 g/cm^3. Assuming the individual layer thicknesses for the β crystal, OAF, and IAF are l_β, l_{OAF}, and l_{IAF}, we then have:

$$L = l_\beta + l_{OAF} + l_{IAF} \quad \text{(Supplementary Eqn. 9)}$$

The weight fraction crystallinity f_β (0.52) can be calculated from the following equation:

$$f_\beta = \frac{l_\beta \rho_\beta}{l_\beta \rho_\beta + l_{OAF}\rho_{OAF} + l_{IAF}\rho_{IAF}} \quad \text{(Supplementary Eqn. 10)}$$

From the D-E loop study in Fig. 2, the minimum f_{OAF} is calculated to be 0.25. Then, we have:

$$f_{OAF} = \frac{l_{OAF}\rho_{OAF}}{l_\beta \rho_\beta + l_{OAF}\rho_{OAF} + l_{IAF}\rho_{IAF}} \geq 0.25 \quad \text{(Supplementary Eqn. 11)}$$

Assuming f_{OAF} is 0.25 (i.e., $P_{s,OAF} = P_{s,\beta}$), we obtain l_{OAF} = 3.02 nm and l_{IAF} = 3.00 nm, and l_β = 5.78 nm. The schematic representation of the three-phase model is shown in Supplementary Fig. 13c with the individual layer thicknesses.

Supplementary Note 11. Molecular dynamics (MD) simulation

Fully atomistic MD simulation was used for the PVDF chains by treating the hydrogen, fluorine and carbon atoms as van der Waals (vdW) monomers with partial electrostatic charges for the carbon atom in the CH_2 group: $q_{C(H)}$ = -0.5202e, for the carbon atom in the CF_2 group: $q_{C(F)}$ =0.6120e, q_H = 0.1807e, and q_F = -0.2266e located at the atomic centers.[11] The monomers of the same chain interact with each other through the polymer specific two-body bond-stretching potential U_{bond}, three-body angle-bending potential U_{angle}, and four-body dihedral angle bending potential $U_{dihedral}$. These potentials are defined as:

$$U_{\text{bond}}(R) = \frac{1}{2} \sum_{\text{all bonds}} k_b (R - R_0)^2 \quad \text{(Supplementary Eqn. 12)}$$

$$U_{\text{angle}}(\theta) = \frac{1}{2} \sum_{\text{all bond pairs}} k_\theta (\theta - \theta_0)^2 \quad \text{(Supplementary Eqn. 13)}$$

$$U_{\text{dihedral}}(\varphi) = \frac{1}{2} \sum_n \sum_{\text{all bond triplets}} k_\varphi(n) \left[1 - \cos(n\varphi)\right] \quad \text{(Supplementary Eqn. 14)}$$

where R_0 and θ_0 are equilibrium (unstretched) bond and angle parameters, k_b, k_θ, and k_φ are the corresponding force constants. All monomer pairs *i* and *j* belonging to different PVDF chains, as well as monomers of the same PVDF chain not involved in the polymeric interactions (Supplementary Eqn. 12-14), have Coulomb and vdW interactions between them,

$$U_q = \sum_{i>j} \frac{q_i q_j e^2}{\varepsilon(r_{ij}) r_{ij}} \quad \text{(Supplementary Eqn. 15)}$$

$$U_{vdW}(r) = \sum_{i>j} A_{ij} \exp(-B_{ij} r_{ij}) - C_{ij}/r_{ij}^6 \quad \text{(Supplementary Eqn. 16)}$$

where r_{ij} is the separation distance between the monomers, A_{ij}, B_{ij} and C_{ij} are atom type specific parameters. The values for these parameters and for the force constants in Supplementary Eqn. 12-14) were taken from the force-field developed by Byutner and Smith for PVDF polymers.[11] The permittivity of the medium $\varepsilon(r)$ in Supplementary Eqn. 15 was considered as a smooth function changing from 1 near a chosen monomer to ε_B = 4 in the bulk, $\varepsilon(r) = 1 + (\varepsilon_B - 1)[(r-1)/(r+1)]$[7].

The simulation box contained 144 PVDF chains oriented parallel to the *z*-axis. Each chain contained 30 $-CH_2CF_2-$ repeat units. Chain ends were attached to the slab walls with rigid C-C bonds. The slab walls, which mimic the surface of the *β* PVDF crystal, were perpendicular to the *z*-axis. The chain attachment points are organized in the same manner as the *ab* unit cell for the β phase of PVDF. The dipoles of the PVDF units at the attachment sites are forced to orient upward along the positive direction of the *y*-axis. The remaining part of the chain was created by

generating random TGTG' conformations along its length. The slab thickness along the *z*-axis was L_z = 6.8 nm. The *ab* unit cell dimensions on the slab wall were a = 2.1 nm and b = 1.2 nm, which were about twice larger than the actual unit cell dimensions of the β crystal (a_0 = 0.86 nm and b_0 = 0.49 nm). Test simulations both with a_0 and b_0, and with a = 1.1 nm and b = 0.6 nm, showed that chain relaxation processes in such highly concentrated systems could not be resolved during the 2-ns long simulation time.

The strain along the *z*-axis was implemented by increasing the slab thickness from L_z to $L_z+\Delta L_z$. After each applied strain, the system was equilibrated for additional 500 ps simulations before the start of the 2-ns long production runs. MD runs with a Langevin thermostat with a friction coefficient γ = 2 ps^{-1} and a Gaussian white-noise force of strength 6kBTγ were performed in a constant NVT ensemble. The equations of motion were integrated using the velocity Verlet algorithm with a time step of 1 fs. We imposed standard periodic boundary conditions by filling space with translational replicas of the fundamental cell in the *x* and *y* directions. The long-range electrostatic interactions between charged particles were handled using the standard Lekner summation algorithm.[12]

References

1. Yang, L., Ho, J., Allahyarov, E., Mu, R. & Zhu, L. Semicrystalline structure dielectric property relationship and electrical conduction in a biaxially oriented poly(vinylidene fluoride) film under high electric fields and high temperatures. *ACS Appl. Mater. Interfaces* **7**, 19894-19905 (2015).
2. Jow, T.R. & Cygan, P.J. Dielectric breakdown of polyvinylidene fluoride and its comparisons with other polymers. *J. Appl. Phys.* **73**, 5147-5151. (1993).
3. Yang, L., Allahyarov, E., Guan, F. & Zhu, L. Crystal orientation and temperature effects on double hysteresis loop behavior in a poly(vinylidene fluoride-*co*-trifluoroethylene-*co*-chlorotrifluoroethylene)-*graft*-polystyrene graft copolymer. *Macromolecules* **46**, 9698-9711 (2013).
4. Li, Y., Ho, J., Wang, J., Li, Z.-M., Zhong, G.-J., Zhu, L. Understanding nonlinear dielectric properties in a biaxially oriented poly(vinylidene fluoride) film at both low and high electric fields. *ACS Appl. Mater. Interfaces* **8**, 455-465 (2016).
5. Furukawa, T., Nakajima, K., Koizumi, T., Date, M. Measurements of nonlinear dielectricity in ferroelectric polymers. *Jpn. J. Appl. Phys.* **26**, 1039-1045 (1987).
6. Feng, S.M., Chai, Y.S., Zhu, J.L., Manivannan, N., Oh, Y.S., Wang, L.J., Yang, Y.S., Jin, C.Q., & Kim, K.H. Determination of the intrinsic ferroelectric polarization in orthorhombic $HoMnO_3$. *New J. Phys.* **12**, 073006 (2010).
7. Stewart, M., Cain, M.G., Direct piezoelectric measurement: The Berlincourt method. In: Cain M. (eds.) *Characterisation of Ferroelectric Bulk Materials and Thin Films*. Springer Series in Measurement Science and Technology, Vol 2. (Springer, Dordrecht, 2014).
8. Dragan Damjanovic. Stress and frequency dependence of the direct piezoelectric effect in ferroelectric ceramics. *J. Appl. Phys.* **82**, 1788-1797 (1997).
9. Barzegar, A. F., Damjanovic, D. & Setter, N. The effect of boundary conditions and sample aspect ratio on apparent d_{33} piezoelectric coefficient determined by direct quasistatic method. *IEEE Trans. Ultrason. Ferroelectr. Freq. Control* **51**, 262-270 (2004).
11. Byutner, O.G. & Smith, G.D. Quantum chemistry based force field for simulations of poly(vinylidene fluoride). *Macromolecules* **33**, 4264-4270 (2000).
12. Mazars, M. Lekner summations. *J. Chem. Phys.* **115**, 2955-2965 (2001).
